# Fine-scale spatial genetic structure of common and declining bumble bees across an agricultural landscape

**DOI:** 10.1111/mec.12823

**Published:** 2014-06-30

**Authors:** Stephanie Dreier, John W Redhead, Ian A Warren, Andrew F G Bourke, Matthew S Heard, William C Jordan, Seirian Sumner, Jinliang Wang, Claire Carvell

**Affiliations:** * Institute of Zoology, Zoological Society of LondonRegent's Park, London, NW1 4RY, UK; † School of Biological Sciences, University of BristolWoodland Road, Bristol, BS8 1UG, UK; ‡ NERC Centre for Ecology & HydrologyMaclean Building, Crowmarsh Gifford, Wallingford, Oxfordshire, OX10 8BB, UK; § School of Biological Sciences, University of East AngliaNorwich Research Park, Norwich, NR4 7TJ, UK

**Keywords:** *Bombus*, conservation, isolation by distance, microsatellite, queen dispersal, relatedness

## Abstract

Land-use changes have threatened populations of many insect pollinators, including bumble bees. Patterns of dispersal and gene flow are key determinants of species' ability to respond to land-use change, but have been little investigated at a fine scale (<10 km) in bumble bees. Using microsatellite markers, we determined the fine-scale spatial genetic structure of populations of four common *Bombus* species (*B. terrestris*, *B. lapidarius*, *B. pascuorum* and *B. hortorum*) and one declining species (*B. ruderatus*) in an agricultural landscape in Southern England, UK. The study landscape contained sown flower patches representing agri-environment options for pollinators. We found that, as expected, the *B. ruderatus* population was characterized by relatively low heterozygosity, number of alleles and colony density. Across all species, inbreeding was absent or present but weak (*F*_IS_ = 0.01–0.02). Using queen genotypes reconstructed from worker sibships and colony locations estimated from the positions of workers within these sibships, we found that significant isolation by distance was absent in *B. lapidarius*, *B. hortorum* and *B. ruderatus*. In *B. terrestris* and *B. pascuorum*, it was present but weak; for example, in these two species, expected relatedness of queens founding colonies 1 m apart was 0.02. These results show that bumble bee populations exhibit low levels of spatial genetic structure at fine spatial scales, most likely because of ongoing gene flow via widespread queen dispersal. In addition, the results demonstrate the potential for agri-environment scheme conservation measures to facilitate fine-scale gene flow by creating a more even distribution of suitable habitats across landscapes.

## Introduction

Land-use change and the consequent loss and degradation of habitats have fragmented the ranges of many insect pollinator species, leading to significant declines in population size and an increased risk of extinction (Potts *et al*. 2010). These patterns are reported worldwide, fuelling ecological and economic concerns over the sustainability of pollination services in the long term (Kremen *et al*. 2002; Cameron *et al*. 2011). Reductions in population size and isolation of previously well-connected populations can decrease the adaptability of organisms to environmental changes through inbreeding and the loss of genetic diversity (Frankham 2005). Declining species therefore tend to show reduced genetic diversity and increased population structuring (Darvill *et al*. 2006; Ellis *et al*. 2006; Charman *et al*. 2010; Cameron *et al*. 2011). In contrast, populations of many common species are connected by high levels of gene flow (Estoup *et al*. 1996; Widmer & Schmid-Hempel 1999). However, few population genetic studies have compared common and declining species within shared landscapes (Lozier *et al*. 2011) and most have been conducted at regional rather than local spatial scales (Jha & Kremen 2013).

Bumble bees (*Bombus* spp.) are important pollinators of a range of native plant species and commercial crops and thus contribute significantly to global crop yields and the persistence of plant communities (Potts *et al*. 2010; Garratt *et al*. 2014). Evidence suggests that they have declined in abundance and range size across Europe and North America in recent decades (Cameron *et al*. 2011). For example, in the UK, seven of the 25 native bumble bee species have suffered serious contractions in range size, although other species remain widespread and apparently abundant (Goulson *et al*. 2008). The causes of bumble bee population declines are likely to be diverse, but, across Europe, a key driver has been agricultural intensification, with increasing loss of habitats and plant species providing key forage resources (Carvell *et al*. 2006; Vanbergen 2013). As many bumble bees nest under or at ground level, nesting sites are also vulnerable to intensive land management practices (Lye *et al*. 2009; Carvell *et al*. 2011).

Bumble bees are also particularly vulnerable to land-use change because, as in other eusocial Hymenoptera, their colony structure and haplodiploid sex determination potentially reduce the effective size of their populations. Bumble bee colonies are typically founded by one, singly-mated queen (Estoup *et al*. 1995; Schmid-Hempel & Schmid-Hempel 2000) and, while colonies can contain more than 100 workers, queens produce all female offspring and nearly all the male offspring. Thus, apparently abundant populations may exhibit limited genetic diversity, making them vulnerable to stochastic genetic and demographic effects (Chapman & Bourke 2001). In small haplodiploid populations, where females are more likely to mate with related males, the likelihood of generating sterile diploid males is increased (Zayed & Packer 2001; Zayed 2004), creating a genetic load associated with reduced colony fitness (Whitehorn *et al*. 2009; Darvill *et al*. 2012). As a result, small and inbred populations of eusocial Hymenoptera, including bumble bees, are at greater risk of extinction.

Patterns of dispersal and gene flow are key determinants of a species' ability to respond to land-use change (Broquet & Petit 2009). Because of haplodiploid sex determination, queens in bumble bees contribute more to gene dispersal than males (Lepais *et al*. 2010). After mating, queens also help disperse male genes as mated queens carry both their own genes and, in stored sperm, those of their mates, when searching to found new colonies. Queen dispersal distances of several kilometres suggest that bumble bee populations are well mixed at fine spatial scales (Lepais *et al*. 2010). However, field observations of where queens found colonies in relation to their natal colonies are very few (Alford 1975; Benton 2006), and there is a lack of information on local (<10 km level) genetic structure in most bumble bees, including declining species (Chapman *et al*. 2003; Jha & Kremen 2013). Conservation options such as agri-environment schemes include among their aims the goal of increasing the connectivity of habitats and populations across landscapes (Natural England 2013). Because landscape connectivity affects gene flow, understanding the local genetic processes associated with population declines is therefore fundamental for the development of effective agri-environment schemes for pollinators. Specifically, there is a need for analyses of fine-scale population structure in intensively managed environments, to determine to what extent these schemes affect landscape connectivity and gene flow in both common and vulnerable species.

We addressed these issues by conducting a genetic study of five social species of bumble bee (*B. terrestris*, *B. lapidarius, B. pascuorum*, *B. hortorum* and *B. ruderatus*) across an agricultural landscape in Southern England, UK. Four of these species are nationally common and widespread, whereas *B. ruderatus* has suffered significant declines in the UK in recent decades and is now restricted to a few sites in southern and central England (NERC 2006). In addition, the widespread *B. hortorum* (Goulson *et al*. 2011) is phylogenetically very closely related to *B. ruderatus* (Cameron *et al*. 2007), providing the opportunity to compare the common and declining members of a phylogenetically close species pair directly. Using microsatellite markers, we characterized levels of genetic diversity, inbreeding and fine-scale patterns of queen dispersal for the five focal species in the study landscape. We sampled the study populations at a fine spatial scale across all potential habitat patches in the landscape to maximize the likelihood of detecting sister workers at multiple sites. This permitted us to estimate the positions of large numbers of colonies and to maximize the proportion of colonies sampled in the landscape. Specifically, we tested the following hypotheses: (i) the declining species (*B. ruderatus*) shows reduced genetic diversity and higher levels of inbreeding than the common species; and (ii) if bumble bee populations are well mixed on a fine scale, then related queens, for example sisters or cousins, do not tend to nest in proximity to one another, leading to an absence of isolation by distance at fine scales. We also assess the implications of our results for the ability of agri-environment schemes to support foraging and nesting behaviours, and promote gene flow, in common and declining bumble bees.

This study adds to previous ones of fine-scale spatial genetic structure in bumble bee populations (Charman *et al*. 2010; Goulson *et al*. 2010; Carvell *et al*. 2012; Jha & Kremen 2013) in two main respects. First, our spatial sampling design based on a two-dimensional grid allows a more detailed resolution than previous approaches to estimating bumble bee colony density or genetic structure which have sampled from spatially independent sites or from points along linear transects. Second, by determining isolation by distance using reconstructed genotypes of queens founding colonies at positions estimated from the spatial distribution of pairs or groups of known sister workers, our study offers a more powerful approach to estimating fine-scale patterns of queen dispersal, intercolony relatedness and gene flow. In particular, this is the first study based on reconstructing queen genotypes and colony locations to address the issue of whether or not related bumble bee queens tend to nest near one another.

## Methods

### Study species

The five study species of *Bombus* vary in their forage plant choice and nesting behaviour. *B. terrestris* and *B. lapidarius* typically nest underground in large colonies and have short-tongued workers that visit a wide range of flowers, whereas *B. pascuorum* and *B. hortorum* tend to live in smaller colonies (on the ground surface in *B. pascuorum* and variously underground, on the surface or above ground in *B. hortorum*) and have longer-tongued workers that specialize in foraging at flowers with long corolla tubes (Benton 2006). *B. ruderatus* is ecologically similar to *B. hortorum*, these being the longest tongued UK *Bombus* species, but the reasons for their contrasting current distribution patterns remain unclear. Studies on *B. ruderatus* have been hampered by its morphological similarity with *B. hortorum*. Hence, for this cryptic species pair, we used a molecular identification method based on mitochondrial DNA markers to allocate samples to their correct species prior to microsatellite genotyping (Ellis *et al*. 2005; Stewart *et al*. 2010; see also Appendix S1, Supporting information). Likewise, we applied a molecular identification method (H.M.G. Lattorff, personal communication) to exclude morphologically similar *B. lucorum* workers from the sample of *B. terrestris* workers.

### Study landscape

**Fig. 1:**
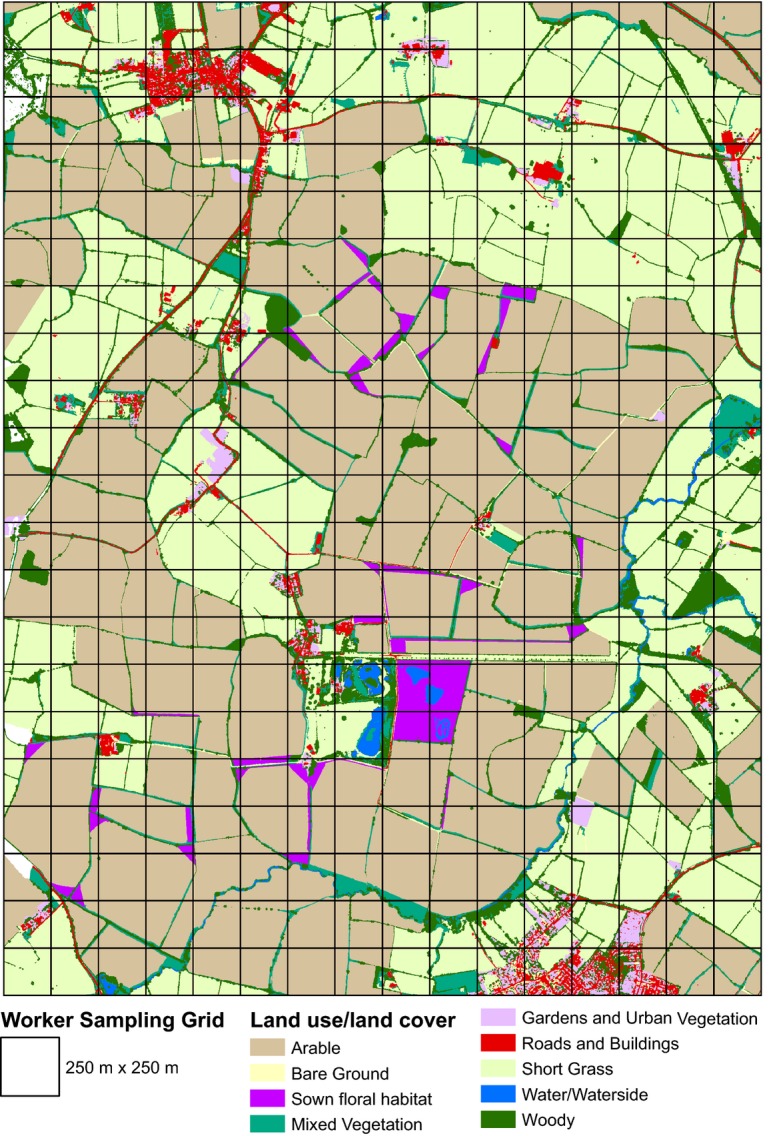
Map of the study landscape in Buckinghamshire, Southern England, UK, showing aggregate land use land cover classes derived from remote sensing data.

The study was conducted across a 1950-ha agricultural landscape, centred on the Hillesden Estate, Buckinghamshire, Southern England (1˚00′01″W; 51˚57′16″N) (Fig. 1). The Estate consists of a c. 1000-ha intensive arable farm on heavy clay soils with a simple rotation of autumn-sown winter wheat *Triticum aestivum*, oilseed rape *Brassica napus* and field beans *Vicia faba*. In 2005, a randomized block experiment was set up to quantify the effects of a then newly introduced UK agri-environment scheme, Entry Level Stewardship (ELS), on biodiversity at the farm scale (Redhead *et al*. 2013). A number of standardized ELS habitat creation options targeted at pollinators, including annual and perennial flower mixtures sown in patches or along field margins, were established alongside conventionally managed fields. Areas of semi-natural habitat such as hedgerows, standard nonsown field margins and trees remained evenly distributed across the farm. The landscape surrounding the Estate is predominantly arable, with some areas of permanent intensive grassland, woodland and small villages.

In August 2007, airborne remote-sensed data were acquired for the entire study landscape. These included light detection and ranging (LiDAR, Optech 3000 ALTM) and hyperspectral [Specim AISA Eagle (400–970 nm)] data. All data sets were geo-referenced and preprocessed. Supervised classification of the hyperspectral data set (erdas imagine v9.0, ERDAS, Georgia, USA), combined with a digital canopy height model derived from LiDAR, produced a high-resolution (0.5 × 0.5 m pixels) map containing 18 land use or land cover (LULC) types including arable crops, intensive grassland, trees and buildings (Fig. 1). Handling of the LULC map, including manual updates to reflect changes in management of sown margins between the collection of remote-sensed data and bumble bee worker sampling, was performed in arcgis (v9.3-10, ESRI, California, USA). For further details on the collection and processing of the LiDAR and hyperspectral data, see Redhead *et al*. (2013).

### Sample collection

The study area was divided into 250 × 250 m grid cells. Within every cell, bumble bee workers were sampled across all potential habitat patches with sampling intensity (i.e. search effort) being broadly proportional to the relative cover of suitable nesting and foraging habitats present. Hence, searches were focussed mainly on field boundaries and other noncrop habitat parcels (defined areas of continuous land use) but also included field centres. Within linear parcels, the full width of the boundary or hedgerow was searched. Within nonlinear parcels, searches focussed within a 6-m-wide transect following a zigzag pattern across the field or woodland. Flowering crops were searched by walking along tramlines and sampling workers within 2 m to either side. Within the few villages or hamlets present, gardens were visited where possible or searches conducted along public rights of way. All workers of the five study *Bombus* species encountered were caught for DNA sampling, and their locations were recorded using a GPS device (accurate to 3 m).

Worker sampling was conducted on 4–5 days per week between 20 June and 5 August 2011. Habitats within each grid cell were searched at least once during this period. Workers were caught and held in a cooled container until a given habitat parcel or grid cell had been searched (to avoid recapturing individuals). The tarsal tip was nonlethally removed from the right mid-leg of each bee (Holehouse *et al*. 2003) and preserved in 100% ethanol until DNA extraction. Sampling was carried out between 09:00 h and 17:00 h during dry weather when ambient temperature was above 11 °C with at least 60% clear sky or above 15 °C under any sky conditions.

### DNA isolation and microsatellite genotyping

DNA was isolated using the HotSHOT protocol (Truett *et al*. 2000). Individuals were genotyped at 10–14 microsatellite loci divided between two or three multiplexes in each case (Estoup *et al*. 1995, 1996; Reber Funk *et al*. 2006; Stolle *et al*. 2009; Tables S1–S5, Supporting information). PCR amplification was carried out in an 8-μL reaction volume containing 4 μL Qiagen multiplex mix, 0.04–0.40 μm of each primer (Tables S1–S5), 0.4 μL dH_2_O and 1.2 μL undiluted DNA template. Amplification conditions involved a HotStarTaq activation step for 15 min at 95 °C followed by 25 cycles of denaturing for 30 s at 94 °C, annealing for 90 s at 57 °C and extension for 1 min at 72 °C; with a final extension of 45 min at 60 °C. Amplified products were visualized on an ABI 3130xl Automated Capillary Sequencer (Applied Biosystems) using a LIZ 500 size standard. Genotypes were resolved using genemapper software v. 4.1.6. All species were genotyped at loci BL03, BL11, BT10, BT26 and BTMS0125. *Bombus terrestris* was additionally typed at BTERN01, BTMS0045, BT18, B96, BTMS0033, BL06, B10, B124 and B126; *B. lapidarius* at BL02, BL06, B10, B11, B131, BTERN02, BTMS0057 and BTMS0136; *B. pascuorum* at BL02, B96, BL06, B10, B124, B126, B131, B132 and BTMS00125; *B. hortorum* at BTERN01, B96, BTMS0045, BT18 and BTMS0136; and *B. ruderatus* at BTERN01, BT18, BTERN02, B131, BTMS0045 and BTMS0136. In total, 2577 workers were successf-ully sampled and genotyped across the five study species.

### Genetic diversity, inbreeding, Hardy–Weinberg equilibrium and linkage disequilibrium

To remove any possible confounding effects of family structure, the sampled workers were reduced by eliminating all but one sibling per inferred family (colony) before the basic population genetic analyses were conducted. Number of alleles (A), observed heterozygosity (H_O_) and expected heterozygosity (H_E_) were calculated in arlequin v. 3.5 (Excoffier *et al*. 2005; Excoffier & Lischer 2010). Effective number of alleles (AE) was calculated in microsoft excel with the poptools version 3.2 add-in. To avoid possible biases caused by using different sets of loci (Tables S1–S5) to make interspecific comparisons in genetic diversity, we recalculated the preceding measures of genetic diversity using only those loci that were genotyped in all the study species (5 homologous loci: BL03, BL11, BTMS0125, BT10 and BT26). Tests for significant inbreeding (significantly greater than zero) were conducted in arlequin on all loci for each species, using 10 000 permutations of gene copies between individuals within populations. This analysis generated population-specific inbreeding coefficients (*F*_IS_) averaged over all loci. Tests for deviation from Hardy–Weinberg equilibrium and linkage disequilibrium were also conducted in arlequin. Hardy–Weinberg *P*-values were obtained using a Markov chain of 100 000 steps. *P*-values for linkage disequilibrium were obtained using Fisher's exact test with 10 000 permutations. Significance levels were adjusted for multiple testing with *P* < 0.05 as appropriate.

### Worker sibship and queen genotype reconstruction

colony version 2.0 (Wang 2004) was used to detect sister relationships among workers. colony implements a full-likelihood approach to sibship analysis, and assignment to these sibships was carried out on the basis of a probability of inference of 0.8 or more. We assumed a monogamous mating system for males and females, therefore allowing the assignment of full-siblings. Male monogamy was assumed as female monogamy and highly male-biased numerical sex ratios among *Bombus* (Bourke 1997; Lopez-Vaamonde *et al*. 2009) suggest that most males mate singly. We carried out a medium run with medium-likelihood precision and a genotyping error rate of 0–5% based on results of regenotyping 10% of randomly selected individuals and scoring errors (Tables S1–S5). The presence of scoring errors was investigated using micro-checker (Van Oosterhout *et al*. 2004). For each analysis, two replicate colony runs were conducted on the same data set, each with a different random number seed. The genotypes of workers in an inferred full-sib group were used to reconstruct the multilocus genotypes of the mother queen for the group.

### Estimating colony locations

The location of each sampled worker was mapped onto the LULC map in arcgis. Colony locations were estimated using a mean centre approach, which involved, first, estimating the colony location as the mean easting and northing of the locations of all workers within a given sibship. The final estimated colony location was then obtained by ‘snapping’ (i.e. moving to coincide exactly with the coordinates of another feature) the mean centre locations to the nearest LULC class that might have formed suitable nesting habitat (i.e. not cropped arable fields, roads, buildings or water). In this case, ‘nearest’ was taken as the closest point on the boundary of the relevant habitat parcel orthogonal to the initially estimated colony location. Alternative approaches tested in preliminary analyses were heavily influenced by either outlying worker locations (e.g. centroid of a minimum convex polygon enclosing all workers in a sibship) or clusters of workers (e.g. median centre). In addition, because the mean centre method involved a purely statistical single-point output requiring no additional parameters or analysis, no prior assumptions regarding likely foraging distances were required. However, the method still yielded similar estimated colony locations to the kernel density estimation method used previously in the same landscape (Carvell *et al*. 2012). Colony locations were estimated only for colonies represented within samples by sibships of two or more workers, as it is not possible to assign a biologically meaningful colony location to colonies represented by a single worker. While colony locations estimated using this approach are undoubtedly subject to error, this is not likely to have been systematic; in other words, the spatial relations of all estimated colony locations within a species should have been reliable estimates of the true pattern of spatial relations. Geographic (Euclidean) distance was calculated between all possible pairs of colonies.

### Isolation by distance

Estimates of relatedness between the reconstructed colony queen genotypes were calculated with coancestry (Wang 2011) following the method of Lynch & Ritland (1999). To determine whether isolation by distance was present within the study landscape, relatedness values between each pair of queens (i.e. mother queens whose genotypes had been reconstructed from the worker sibships) were plotted against the log._10_-transformed geographic distance between the estimated locations of the colonies founded by these queens. The significance of the correlation was calculated with a Mantel test implemented in r v. 3.0.0 (R Development Core Team 2012) using the package ‘vegan’. *P*-values were computed using the negative tail as tests were conducted between similarity and dissimilarity matrices.

## Results

### Hardy–Weinberg equilibrium and linkage disequilibrium

Null alleles and stutter peaks were detected at 1–4 loci per species. However, we did not find null alleles consistently for the same loci across species (Tables S1–S5), suggesting no systematic biases in PCR amplification. Moreover, null alleles did not contribute to significant homozygote excess as no locus deviated significantly from Hardy–Weinberg equilibrium following correction for multiple testing (Tables S1–S5). In addition, none of the loci in any of the study species showed evidence of allelic dropout. Significant linkage disequilibrium (after correction for multiple testing) was detected in only 1 of 55 pairwise comparisons, between the loci BTERN01 and B131 in *B. ruderatus*. No significant linkage disequilibrium was detected between these two loci in any of the other species. Thus, we concluded that genuine linkage disequilibrium between BTERN01 and B131 is absent.

### Genetic diversity and inbreeding

Across all the study species, the number of alleles per locus (A) varied from 10.55 to 19.50, and the effective number of alleles (AE) from 4.39 to 9.55 (Table 1). Observed heterozygosity ranged from 0.66 to 0.83 and expected heterozygosity from 0.67 to 0.84 (Table 1). The declining species *B. ruderatus* had the second lowest observed heterozygosity, the lowest number of alleles and the second lowest effective number of alleles among the study species; by contrast, the closely related *B. hortorum* had the highest observed heterozygosity, the highest number of alleles and the highest effective number of alleles (Table 1). When comparisons were made using only the 5 homologous loci, the results were very similar in that *B. ruderatus* again had the second lowest observed heterozygosity, the lowest number of alleles and the second lowest effective number of alleles, whereas *B. hortorum* had the second highest observed heterozygosity, the second highest number of alleles and the highest effective number of alleles (Table 1). Four of the study species (*B. terrestris*, *B. lapidarius*, *B. pascuorum* and *B. hortorum*) exhibited significantly positive F_IS_ values, whereas *B. ruderatus* exhibited a marginally significantly positive *F*_IS_ value (Table 1). However, *F*_IS_ values were consistently low (0.01–0.02) and, given the lack of deviation of the populations from Hardy–Weinberg equilibrium, it appears that inbreeding was either absent from the study populations or, if present, very weak.

**Table 1:** Population genetic parameters from the analysis of microsatellite genotypes of workers of five *Bombus* species in the study landscape, based on all loci (upper part of the table; worker sample sizes as in Table 2) and based on the 5 homologous loci genotyped in all species (lower part of the table; worker sample sizes standardized to 88 workers in each species, corresponding to the lowest sample size for any single species, which was in *B. ruderatus*)

Species	No. of loci	H_O_	H_E_	A	AE	*F*_IS_	*P*
All loci							
*B. terrestris*	14	0.79 (0.025)	0.81 (0.025)	15.50 (2.511)	6.21 (0.709)	0.020 (0.009)	0.003
*B. lapidarius*	13	0.74 (0.029)	0.74 (0.027)	11.23 (1.311)	4.39 (0.415)	0.011 (0.005)	0.019
*B. pascuorum*	14	0.66 (0.058)	0.67 (0.062)	12.07 (2.205)	4.60 (0.904)	0.013 (0.010)	0.039
*B. hortorum*	10	0.83 (0.042)	0.84 (0.040)	19.50 (2.738)	9.55 (1.898)	0.017 (0.015)	0.018
*B. ruderatus*	11	0.73 (0.032)	0.75 (0.032)	10.55 (0.976)	4.56 (0.551)	0.023 (0.015)	0.071
Homologous loci							
*B. terrestris*	5	0.85 (0.018)	0.87 (0.016)	17.40 (3.682)	7.69 (0.851)		
*B. lapidarius*	5	0.74 (0.041)	0.76 (0.043)	9.60 (1.720)	4.66 (0.805)		
*B. pascuorum*	5	0.78 (0.061)	0.79 (0.061)	14.40 (3.957)	6.62 (1.961)		
*B. hortorum*	5	0.79 (0.090)	0.80 (0.074)	16.20 (3.720)	9.04 (3.291)		
*B. ruderatus*	5	0.76 (0.047)	0.76 (0.045)	9.20 (1.393)	4.82 (0.907)		

H_O_, mean (SE) observed heterozygosity; H_E_, mean (SE) expected heterozygosity; A, mean (SE) number of alleles; AE, mean (SE) effective number of alleles; *F*_IS_, inbreeding coefficient (SE); *P*, significance values from tests of the *F*_IS_ values against zero.

### Queen genotype reconstruction analysis

The genotypes of 88–668 queens per species were reconstructed by colony from the genotypes of worker sibships (Table 2). Of these, 52–75% were reconstructed from only one worker. The probabilities of inference for these genotypes were low (0.19–0.38; Fig. S1, Supporting information), as expected given that one worker provides just 50% information about its maternal genotype. Therefore, queen genotypes reconstructed from single workers were excluded from the analysis of isolation by distance. Error in the remaining queen genotype reconstructions is unlikely to have affected our results regarding isolation by distance, as there is no reason to expect this error to vary systematically with geographic position. Following implementation of these methods, the numbers of reconstructed queen genotypes ranged across the study species from 42 to 271, based on worker sibships of mean sizes 2.44–4.00 and a probability of inference of 0.57–0.75 (Table 2). Estimated minimum colony densities per species across the study landscape varied from 2.2 to 14.3 colonies km^−2^ when colonies represented by single workers were excluded and from 4.6 to 35.2 km^−2^ when all sampled colonies were included (Table 3). *B. ruderatus* exhibited the lowest estimates of minimum colony density (Table 3).

**Table 2:** Sample sizes (numbers of workers genotyped, size of worker sibships and number of reconstructed queen genotypes) and mean probability of inference of the reconstructed queen genotypes for the five *Bombus* species in the study landscape

Species	Total no. of workers genotyped*	No. of workers used for genetic diversity analyses†	Mean (range) no. of workers within sibships‡	No. of reconstructed queen genotypes*	Probability of inference (± SE) of reconstructed queen genotypes‡
*B. terrestris*	187 (382)	264	2.71 (2–8)	69 (264)	0.66 ± 0.018
*B. lapidarius*	774 (1171)	668	2.86 (2–11)	271 (668)	0.75 ± 0.008
*B. pascuorum*	311 (548)	360	2.53 (2–7)	123 (360)	0.71 ± 0.012
*B. hortorum*	117 (262)	193	2.44 (2–6)	48 (193)	0.57 ± 0.027
*B. ruderatus*	168 (214)	88	4.00 (2–19)	42 (88)	0.74 ± 0.026
Total	1557 (2577)	1573		553 (1573)	

* From colonies with >1 assigned worker and, in parentheses, from all colonies.

† One individual per colony.

‡ For colonies with >1 assigned worker only.

**Table 3:** Estimated minimum colony densities for five species of *Bombus* in the study landscape

	Minimum colony density (colonies km^−2^)	
	
	Estimated from colonies with >1 assigned worker	Estimated from all colonies
*B. terrestris*	3.63	13.89
*B. lapidarius*	14.26	35.16
*B. pascuorum*	6.47	18.95
*B. hortorum*	2.53	10.16
*B. ruderatus*	2.21	4.63

### Isolation by distance

**Fig. 2:**
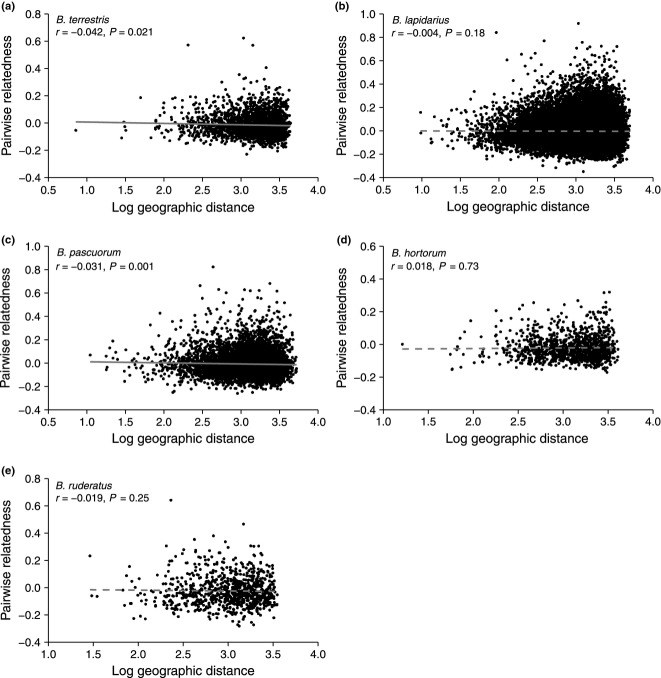
Relationship between pairwise relatedness of colony queens (whose genotypes were reconstructed from worker sibships) and geographic distance (log._10_-transformed distance in metres) between colonies in populations of (a) *Bombus terrestris*, (b) *B. lapidarius*, (c) *B. pascuorum*, (d) *B. hortorum* and (e) *B. ruderatus* in the study landscape. Results of the Mantel tests are reported on the plots. Plain and dashed lines represent significant and nonsignificant correlations, respectively. Regression equations: *B. terrestris*, *y* = −0.0101x + 0.0172, *B. lapidarius*, *y* = −0.0014x + 0.0006, *B. pascuorum*, *y* = −0.0093x + 0.0207, *B. hortorum*, *y* = 0.0037*x* – 0.0326 and *B. ruderatus*, *y* = −0.0059*x* – 0.0068. Sample sizes (no. of reconstructed queen genotypes) are as in Table 2.

Within the study landscape, two species (*B. terrestris* and *B. pascuorum*) exhibited significant isolation by distance, with pairwise relatedness between colony queens decreasing as intercolony geographic distance increased (Mantel test: *B. terrestris*, *r* = −0.042, *P* = 0.021; *B. pascuorum*, *r* = −0.031, *P* = 0.001; Fig. 2a and c). The three other species showed no significant relationship between pairwise relatedness of colony queens and intercolony geographic distance (Fig. 2b, d and e). However, in *B. terrestris* and *B. pascuorum*, the overall pattern of the relationship between pairwise relatedness of colony queens and intercolony geographic distance was very similar to that of the other three species (Fig. 2), and geographic distance explained only a very small proportion of the variance in pairwise relatedness of colony queens (*r*^2^ = 0.002 and 0.001, respectively). In addition, estimated relatedness between close neighbours was very low and declined to zero over a relatively short distance. For example, in *B. terrestris*, if two queens had founded colonies 1 m apart, their expected pairwise relatedness would have been 0.02 (estimated from the equation of the relationship between relatedness and log._10_ geographic distance in Fig. 2a). The expected pairwise relatedness between queens would fall to zero once colonies were only 50.5 m apart (again estimated from the regression equation in Fig. 2a). The sample mean pairwise relatedness values (R) were all very close to zero (mean ± SE: *B. terrestris*, *R* = −0.01 ± 0.002; *B. lapidarius*, *R* = 0.00 ±0.001; *B. pascuorum*, *R* = −0.01 ± 0.001; *B. hortorum*, *R* = −0.02 ± 0.002; *B. ruderatus*, *R* = −0.02 ± 0.004). In *B. terrestris*, the expected pairwise relatedness falls to the sample mean at a colony separation distance of only 493 m. These findings suggest that isolation by distance in *B. terrestris* and *B. pascuorum*, if present, is very weak.

## Discussion

We conducted population genetic analyses of the fine-scale spatial structure of four widespread and one declining bumble bee species occurring sympatrically within an agricultural landscape. Specifically, we tested the hypotheses that (i) the declining species (*B. ruderatus*) shows reduced genetic diversity and higher levels of inbreeding than the common species; and (ii) related queens do not tend to nest in proximity to one another at fine spatial scales. We found that, in all species, inbreeding was either absent or, if present, extremely weak. In terms of genetic diversity, we found that, in contrast to the closely related *B. hortorum*, the declining species *B. ruderatus* had the second lowest observed heterozygosity, the lowest allelic diversity and the second lowest effective number of alleles among the study species. The results therefore support our first hypothesis with respect to genetic diversity but fail to demonstrate greater inbreeding levels in the declining species relative to those shown by the common species at the study site. The results also support the second hypothesis, because we found that isolation by distance was either absent or only very weakly present. Together, these results suggest that gene flow in these populations of common and declining bumble bees is unconstrained at a fine spatial scale. In particular, they show that, in agricultural landscapes at this scale, queen dispersal and settlement patterns are such that bumble bee colonies nesting near one another are essentially unrelated and hence that populations are well mixed.

### Genetic diversity and inbreeding

Our finding that the declining species *B. ruderatus* tended to exhibit low genetic diversity (as measured by observed heterozygosity, allelic diversity and the effective number of alleles) is consistent with previous results showing an association in bumble bees between population decline and a reduction in levels of genetic variation (Ellis *et al*. 2006; Goulson *et al*. 2008; Charman *et al*. 2010; Lozier *et al*. 2011; but see Lozier 2014). However, stronger conclusions are not possible from our data because only one population per species was studied. The general lack of substantial inbreeding (range of *F*_IS_ = 0.01–0.02) was likely to have stemmed from the absence of obvious physical barriers to queen and male premating dispersal within the study landscape. In other populations of *B. terrestris* and *B. pascuorum* within agricultural and urban habitats, no evidence of inbreeding has been found (Chapman *et al*. 2003; Darvill *et al*. 2004).

### Colony density and isolation by distance

From worker sibship analyses, we were able to estimate the minimum densities of colonies at the study site. *B. ruderatus* had the lowest minimum colony density of any of the five study species, despite initially similar worker abundance to the closely related species, *B. hortorum* (Tables 2 and 3). *B. lapidarius* had the highest minimum colony density and *B. pascuorum* the second highest (Table 3). These findings are consistent with the restricted distribution and declining population status of *B. ruderatus* and suggest that low colony density may contribute to relatively low genetic diversity in this species. The findings are also consistent with other studies that have found *B. lapidarius* and *B. pascuorum* to exhibit high colony densities in UK agricultural habitats (Darvill *et al*. 2004; Knight *et al*. 2005).

The lack of a substantial relationship between intercolony queen relatedness and geographic distance in all five species shows that, at the scale of the study landscape, bumble bee queens do not tend to found colonies close to related queens; this must stem from relatively extensive dispersal of queens between departure from the natal colony and colony foundation. This conclusion is consistent with previous findings of queen dispersal distances of several kilometres (Lepais *et al*. 2010) and implies that queens sampled in the study landscape included both queens reared within the landscape and those immigrating to it from surrounding areas.

The processes driving genetic structure are likely to be complex. In bumble bees, fine-scale spatial genetic structure almost certainly stems from the combined effects of gene flow, effective population size and environmental factors such as landscape structure and habitat fragmentation (Goulson *et al*. 2011; Jha & Kremen 2013; Lozier *et al*. 2013). We suggest high levels of gene flow as a partial explanation of the absent or weak isolation by distance found at the fine scale among the sampled bumble bee populations, although comparative studies in contrasting landscapes at different spatial scales could prove valuable to further elucidate the effects of landscape structure on population genetic structure. Consistent with our current data, analyses of *B. lapidarius* and *B. pascuorum* workers collected from the same study landscape during 2009 showed no significant genetic differentiation among or between samples (Carvell *et al*. 2012). However, bumble bee populations may vary with respect to fine-scale spatial genetic structuring. In a recent study of *B. vosnesenskii* in North America, Jha & Kremen (2013) found evidence of significant fine-scale spatial genetic structure between colonies at the 1–9 km spatial scale. This study was based on workers sampled at two scales (at a fine scale along linear transects separated at larger scales) and not, as is the current study, on queen genotypes and colony locations reconstructed from worker sibships sampled at a fine spatial scale across a two-dimensional grid. At larger spatial scales, regional-level or continental-level population genetic differentiation is typically weak or absent in widespread bumble bee species (Widmer & Schmid-Hempel 1999; Chapman *et al*. 2003; Lozier *et al*. 2011) and more marked in declining species or populations occupying physically separated environments such as groups of islands (Darvill *et al*. 2006; Ellis *et al*. 2006; Charman *et al*. 2010; Goulson *et al*. 2011; Lozier *et al*. 2011).

Finally, our results potentially inform conservation management for bumble bees. Jha & Kremen's (2013) finding that *B*. *vosnesenskii* exhibited significant fine-scale spatial genetic structure may have arisen from methodological differences compared with our study. However, it may reflect more the fact that their study area had recently undergone expansions in agriculture and urbanization, which in turn limited queen dispersal. Our study landscape featured restored habitats in the form of sown flower mixtures within the intensive agricultural matrix. These mixtures created high-value foraging and nesting resources at spatial scales within the likely foraging distance of most *Bombus* species (in some areas occupying >3% of farmed land area). Such targeted agri-environment conservation measures have been shown to increase bumble bee abundance and potentially reduce worker foraging distances (Carvell *et al*. 2011, 2012), but the ability of these measures to promote dispersal and gene flow has been unknown. By showing an overall lack of fine-scale spatial genetic structure, or the presence of at most very weak structure, our findings suggest that a typical agricultural landscape enhanced by agri-environment measures does not present substantial barriers to queen dispersal or gene flow in bumble bees.

## Data accessibility

Microsatellite genotype data for five species of bumblebee across an agricultural landscape in Buckinghamshire, UK: NERC Environmental Information Data Centre CEH:EIDC:1401189757278; doi:10.5285/6a408415-0575-49c6-af69-b568e343266d.

Geographic distances between pairs of wild bumblebee colonies across an agricultural landscape in Buckinghamshire, UK: NERC Environmental Information Data Centre CEH:EIDC:1401193864362; doi:10.5285/8b3f4857-9809-43cb-b2aa-a988e64a5449.

## Supporting information

**Appendix S1** Methods. **Table S1** Description of the microsatellite loci used to genotype *B. terrestris* workers. **Table S2** Description of the microsatellite loci used to genotype *B. lapidarius* workers. **Table S3** Description of the microsatellite loci used to genotype *B. pascuorum* workers. **Table S4** Description of the microsatellite loci used to genotype *B. hortorum* workers. **Table S5** Description of the microsatellite loci used to genotype *B. ruderatus* workers. **Fig. S1** Probability of inferring the mother queen's genotype as a function of the number of worker offspring in a given sibship in the five *Bombus* study species.
