# Supplementary material for: Fine-scale spatial genetic structure of common and declining bumble bees across an agricultural landscape
Source: Mol Ecol. 2014 Jun 30;23(14):3384–95. doi: 10.1111/mec.12823 (PMC4142012; doi:10.1111/mec.12823)
Supplement: Supplementary file 1 [file mec0023-3384-SD1.docx]

**Fine-scale spatial genetic structure of common and declining bumble bees across an agricultural landscape: Supporting Information**

STEPHANIE DREIER,*† JOHN W. REDHEAD,‡ IAN WARREN,*† ANDREW F. G. BOURKE,§ MATTHEW S. HEARD,‡ WILLIAM C. JORDAN,* SEIRIAN SUMNER,*† JINLIANG WANG,* and CLAIRE CARVELL‡

**Institute of Zoology, Zoological Society of London, Regent's Park, London NW1 4RY, UK*

†*School of Biological Sciences, University of Bristol, Bristol BS8 1UG, UK*

‡*NERC Centre for Ecology & Hydrology, Maclean Building, Crowmarsh Gifford,*

*Wallingford, Oxfordshire OX10 8BB, UK*

§*School of Biological Sciences, University of East Anglia, Norwich Research Park, Norwich NR4 7TJ, UK*

Correspondence: Stephanie Dreier, E-mail: [stephaniedreier21@gmail.com](mailto:stephaniedreier21@gmail.com)

**Supporting Information – Methods**

*Molecular test for separating* Bombus ruderatus *and* B. hortorum *workers*

The method of Stewart *et al.* (2010) was applied to distinguish *B. ruderatus* workers from *B. hortorum* workers. The method combines two non-specific external primers and a species-specific internal primer. The *cytochrome b* fragment (426 bp) is amplified using Ellis *et al*’s (2005) primers CYTBF2 and CYTBR2, and the additional internal primer BHR1, specific to *B. hortorum*, amplifies a short section (255 bp) of the gene if the DNA is from *B. hortorum*. The PCR conditions involved a 14.81 μl total reaction volume containing 7 μl Qiagen multiplex mix, 1.43 μl of 3μM primer CYTBF, 1.43 μl of 3 μM primer CYTBR, 1.95 μl of 0.2 μM primer BHR1, 2.5 μl dH_2_O and 0.5 μl template DNA. The PCR involved a HotStarTaq activation step for 15 min at 95ºC followed by 25 cycles of denaturing for 30 s at 94ºC, annealing for 1 min at 48ºC, extension for 1 min at 72ºC; with a final extension of 30 min at 60ºC. The PCR product was run on a 1% agarose gel, yielding a single band of 426 bp in the case of *B. ruderatus* and one of 255 bp in the case of *B. hortorum*. It did not prove possible to amplify both the 426 bp and 255 bp fragments in the *B. hortorum* samples, possibly due to the fact that the internal primer BHR1 and generic forward primer CYTBF2 were preferentially amplifying over CYTBF2/CYTBR2. The 426 bp fragment is essentially a positive control, showing that *B. ruderatus* DNA has amplified.

**Table S1** Description of the microsatellite loci used to genotype *B. terrestris* workers. Sources: (a) Reber Funk *et al.* (2006); (b) Stolle *et al.* (2009); (c) Estoup *et al.* (1995, 1996). Concn: final primer concentration in the PCR; H_O_: observed heterozygosity; H_E_: expected heterozygosity; A: number of alleles; F_IS_: inbreeding coefficient; *P*: *P*-value (exact tests) for departure from Hardy-Weinberg equilibrium; Error rate: genotyping error rate; ^*^null alleles detected.

| **Primer Set** | **Locus** | **Source** | **Dye** | **Range (bp)** | **Concn (µM)** | **H_O_** | **H_E_** | **A** | **F_IS_** | **P** | **Error rate** |
| --- | --- | --- | --- | --- | --- | --- | --- | --- | --- | --- | --- |
| A | BTERN01 | a | 6-FAM | 93-119 | 0.10 | 0.773 | 0.760 | 12 | -0.0166 | 0.2781 | 0.00 |
| A | BT10 | a | 6-FAM | 146-182 | 0.10 | 0.845 | 0.834 | 16 | -0.0134 | 0.0343 | 0.00 |
| A | BTMS0045 | b | 6-FAM | 230-250 | 0.10 | 0.787 | 0.790 | 11 | 0.0043 | 0.2244 | 0.01 |
| A | BT26 | a | VIC | 110-214 | 0.32 | 0.838 | 0.900 | 44 | 0.0686 | 0.2995 | 0.05 * |
| A | BL11 | a | PET | 148-186 | 0.40 | 0.780 | 0.796 | 17 | 0.0201 | 0.0831 | 0.02 |
| B | BT18 | a | 6-FAM | 172-192 | 0.12 | 0.695 | 0.740 | 9 | 0.0618 | 0.5030 | 0.05 * |
| B | B96 | c | 6-FAM | 234-242 | 0.12 | 0.531 | 0.551 | 4 | 0.0367 | 0.4040 | 0.00 |
| B | BL03 | a | VIC | 125-159 | 0.04 | 0.866 | 0.878 | 15 | 0.0138 | 0.9826 | 0.00 |
| B | BTMS0033 | b | VIC | 194-217 | 0.12 | 0.760 | 0.738 | 7 | -0.0303 | 0.1728 | 0.00 |
| B | BL06 | a | NED | 144-172 | 0.10 | 0.760 | 0.797 | 14 | 0.0476 | 0.0706 | 0.00 |
| C | B10 | c | 6-FAM | 177-217 | 0.16 | 0.863 | 0.909 | 21 | 0.0504 | 0.2118 | 0.00 |
| C | B124 | c | 6-FAM | 235-271 | 0.08 | 0.890 | 0.882 | 18 | -0.0086 | 0.9520 | 0.00 |
| C | BTMS0125 | b | VIC | 100-122 | 0.08 | 0.902 | 0.898 | 12 | -0.0037 | 0.7741 | 0.00 |
| C | B126 | c | VIC | 154-204 | 0.08 | 0.790 | 0.828 | 17 | 0.0460 | 0.0233 | 0.05* |

**Table S2** Description of the microsatellite loci used to genotype *B. lapidarius* workers. Sources: (a) Reber Funk *et al.* (2006); (b) Stolle *et al.* (2009); (c) Estoup *et al.* (1995, 1996). Concn: final primer concentration in the PCR; H_O_: observed heterozygosity; H_E_: expected heterozygosity; A: number of alleles; F_IS_: inbreeding coefficient; *P*: *P*-value (exact tests) for departure from Hardy-Weinberg equilibrium; Error rate: genotyping error rate; ^*^null alleles detected.

| **Primer Set** | **Locus** | **Source** | **Dye** | **Range (bp)** | **Concn (µM)** | **H_O_** | **H_E_** | **A** | **F_IS_** | **P** | **Error rate** |
| --- | --- | --- | --- | --- | --- | --- | --- | --- | --- | --- | --- |
| A | BL02 | a | 6-FAM | 149-159 | 0.2 | 0.728 | 0.726 | 6 | -0.0021 | 0.8754 | 0.00 |
| A | BL03 | a | VIC | 130-174 | 0.2 | 0.684 | 0.677 | 20 | -0.0097 | 0.9635 | 0.00 |
| A | BL06 | a | NED | 154-188 | 0.2 | 0.759 | 0.793 | 18 | 0.0424 | 0.2037 | 0.00 |
| A | BL11 | a | PET | 131-149 | 0.2 | 0.855 | 0.850 | 10 | -0.0054 | 0.3499 | 0.00 |
| B | B10 | c | 6-FAM | 202-226 | 0.4 | 0.792 | 0.793 | 12 | 0.0015 | 0.2189 | 0.00 |
| B | BTMS0125 | b | VIC | 96-116 | 0.2 | 0.750 | 0.768 | 11 | 0.0237 | 0.4614 | 0.00 |
| B | B11 | c | VIC | 141-187 | 0.2 | 0.813 | 0.821 | 12 | 0.0100 | 0.9919 | 0.05 |
| B | B131 | c | NED | 133-143 | 0.2 | 0.561 | 0.586 | 6 | 0.0435 | 0.5577 | 0.00* |
| B | BTERN02 | a | PET | 152-180 | 0.4 | 0.780 | 0.793 | 15 | 0.0171 | 0.0109 | 0.00 |
| C | BTMS0057 | b | 6-FAM | 115-137 | 0.2 | 0.808 | 0.805 | 12 | -0.0043 | 0.9720 | 0.00 |
| C | BT10 | a | 6-FAM | 90-164 | 0.2 | 0.856 | 0.854 | 13 | -0.0025 | 0.0641 | 0.00 |
| C | BT26 | a | VIC | 103-145 | 0.2 | 0.630 | 0.637 | 7 | 0.0108 | 0.9575 | 0.00 |
| C | BTMS0136 | b | VIC | 145-151 | 0.2 | 0.542 | 0.558 | 4 | 0.0295 | 0.5732 | 0.00 |

**Table S3** Description of the microsatellite loci used to genotype *B. pascuorum* workers. Sources: (a) Reber Funk *et al.* (2006); (b) Stolle *et al.* (2009); (c) Estoup *et al.* (1995, 1996). Concn: final primer concentration in the PCR; H_O_: observed heterozygosity; H_E_: expected heterozygosity; A: number of alleles; F_IS_: inbreeding coefficient; *P*: *P*-value (exact tests) for departure from Hardy-Weinberg equilibrium; Error rate: genotyping error rate; ^*^null alleles detected.

| **Primer Set** | **Locus** | **Source** | **Dye** | **Range (bp)** | **Concn (µM)** | **H_O_** | **H_E_** | **A** | **F_IS_** | **P** | **Error rate** |
| --- | --- | --- | --- | --- | --- | --- | --- | --- | --- | --- | --- |
| A5 | BL02 | a | 6-FAM | 147-155 | 0.2 | 0.700 | 0.682 | 5 | -0.0268 | 0.6466 | 0.00 |
| A5 | B96 | c | VIC | 210-240 | 0.2 | 0.711 | 0.719 | 12 | 0.0117 | 0.5557 | 0.00 |
| A5 | BL03 | a | VIC | 124-156 | 0.2 | 0.825 | 0.818 | 16 | -0.0089 | 0.0112 | 0.00 |
| A5 | BL06 | a | NED | 145-149 | 0.2 | 0.331 | 0.312 | 3 | -0.0588 | 0.6084 | 0.00 |
| A5 | BL11 | a | PET | 119-133 | 0.3 | 0.625 | 0.642 | 7 | 0.0262 | 0.6461 | 0.00 |
| B5 | B10 | c | 6-FAM | 169-179 | 0.2 | 0.117 | 0.111 | 5 | -0.0522 | 1.0000 | 0.00 |
| B5 | B124 | c | 6-FAM | 245-265 | 0.2 | 0.739 | 0.758 | 9 | 0.0251 | 0.7243 | 0.00 |
| B5 | B126 | c | VIC | 124-132 | 0.4 | 0.457 | 0.441 | 5 | -0.0361 | 0.7202 | 0.00 |
| B5 | B131 | c | NED | 118-148 | 0.2 | 0.772 | 0.793 | 15 | 0.0265 | 0.8267 | 0.00 |
| B5 | B132 | c | PET | 144-174 | 0.3 | 0.772 | 0.783 | 15 | 0.0138 | 0.1253 | 0.00 |
| C5 | BT10 | a | 6-FAM | 114-156 | 0.2 | 0.875 | 0.900 | 20 | 0.0275 | 0.5489 | 0.00 |
| C5 | BTMS0045 | b | 6-FAM | 213-245 | 0.2 | 0.745 | 0.807 | 16 | 0.0768 | 0.2356 | 0.05* |
| C5 | BT26 | a | VIC | 100-112 | 0.2 | 0.684 | 0.673 | 7 | -0.0166 | 0.2383 | 0.02 |
| C5 | BTMS0125 | b | VIC | 129-197 | 0.2 | 0.894 | 0.928 | 34 | 0.0363 | 0.1717 | 0.02* |

**Table S4** Description of the microsatellite loci used to genotype *B. hortorum* workers. Sources: (a) Reber Funk *et al.* (2006); (b) Stolle *et al.* (2009); (c) Estoup *et al.* (1995, 1996). Concn: final primer concentration in the PCR; H_O_: observed heterozygosity; H_E_: expected heterozygosity; A: number of alleles; F_IS_: inbreeding coefficient; *P*: *P*-value (exact tests) for departure from Hardy-Weinberg equilibrium; Error rate: genotyping error rate; ^*^null alleles detected.

| **Primer Set** | **Locus** | **Source** | **Dye** | **Range (bp)** | **Concn (µM)** | **H_O_** | **H_E_** | **A** | **F_IS_** | **P** | **Error rate** |
| --- | --- | --- | --- | --- | --- | --- | --- | --- | --- | --- | --- |
| A | BTERN01 | a | 6-FAM | 91-159 | 0.15 | 0.922 | 0.937 | 29 | 0.0162 | 0.4074 | 0.00 |
| A | B96 | c | 6-FAM | 221-251 | 0.15 | 0.845 | 0.844 | 13 | -0.0007 | 0.3280 | 0.02 |
| A | BTMS0045 | b | 6-FAM | 280-322 | 0.55 | 0.921 | 0.926 | 22 | 0.0053 | 0.3675 | 0.02 |
| A | BT26 | a | VIC | 105-131 | 0.15 | 0.876 | 0.881 | 14 | 0.0062 | 0.0537 | 0.00 |
| A | BL03 | a | VIC | 134-144 | 0.08 | 0.637 | 0.604 | 6 | -0.0559 | 0.9536 | 0.00 |
| B | BT10 | a | 6-FAM | 103-189 | 0.20 | 0.948 | 0.957 | 37 | 0.0095 | 0.6875 | 0.00 |
| B | BT18 | a | 6-FAM | 180-218 | 0.20 | 0.843 | 0.884 | 18 | 0.0460 | 0.4306 | 0.00 |
| B | BTMS0125 | b | VIC | 86-128 | 0.10 | 0.560 | 0.621 | 19 | 0.0998 | 0.1467 | 0.05* |
| B | BTMS0136 | b | VIC | 142-176 | 0.20 | 0.764 | 0.839 | 16 | 0.0894 | 0.3366 | 0.05* |
| B | BL11 | a | PET | 109-149 | 0.40 | 0.943 | 0.910 | 21 | -0.0355 | 0.3381 | 0.00 |

**Table S5** Description of the microsatellite loci used to genotype *B. ruderatus* workers. Sources: (a) Reber Funk *et al.* (2006); (b) Stolle *et al.* (2009); (c) Estoup *et al.* (1995, 1996). Concn: final primer concentration in the PCR; H_O_: observed heterozygosity; H_E_: expected heterozygosity; A: number of alleles; F_IS_: inbreeding coefficient; *P*: *P*-value (exact tests) for departure from Hardy-Weinberg equilibrium; Error rate: genotyping error rate; ^*^null alleles detected.

| **Primer Set** | **Locus** | **Source** | **Dye** | **Range (bp)** | **Concn (µM)** | **H_O_** | **H_E_** | **A** | **F_IS_** | **P** | **Error rate** |
| --- | --- | --- | --- | --- | --- | --- | --- | --- | --- | --- | --- |
| A | BTERN01 | a | 6-FAM | 95-131 | 0.20 | 0.739 | 0.703 | 14 | -0.0508 | 0.8284 | 0.00 |
| A | BT10 | a | 6-FAM | 135-155 | 0.10 | 0.818 | 0.852 | 11 | 0.0401 | 0.9342 | 0.02* |
| A | BT18 | a | 6-FAM | 184-218 | 0.15 | 0.795 | 0.800 | 8 | 0.0060 | 0.8226 | 0.02* |
| A | BT26 | a | VIC | 95-125 | 0.15 | 0.750 | 0.746 | 10 | -0.0051 | 0.4572 | 0.00 |
| A | BL03 | a | VIC | 143-165 | 0.10 | 0.750 | 0.732 | 9 | -0.0243 | 0.8011 | 0.00 |
| A | BTERN02 | a | VIC | 177-215 | 0.35 | 0.693 | 0.702 | 15 | 0.0120 | 0.5333 | 0.02* |
| B | B131 | c | 6-FAM | 134-148 | 0.15 | 0.489 | 0.531 | 8 | 0.0802 | 0.3329 | 0.00 |
| B | BTMS0045 | b | 6-FAM | 282-332 | 0.40 | 0.773 | 0.866 | 14 | 0.1086 | 0.0032 | 0.05* |
| B | BTMS0125 | b | VIC | 102-110 | 0.15 | 0.602 | 0.623 | 4 | 0.0341 | 0.5970 | 0.00 |
| B | BTMS0136 | b | VIC | 144-174 | 0.30 | 0.750 | 0.811 | 11 | 0.0756 | 0.0252 | 0.00 |
| B | BL11 | a | PET | 125-149 | 0.45 | 0.885 | 0.869 | 12 | -0.0184 | 0.5511 | 0.00 |

**Fig. S1** Probability of inferring the mother queen's genotype as a function of the number of worker offspring in a given sibship in the five *Bombus* study species. The inference was obtained in a likelihood framework by calculating the probability of observing the genotypes of the offspring of an inferred queen (Wang 2004). bhor = *B. hortorum*, blap = *B. lapidarius*, bpas = *B. pascuorum*, brud = *B. ruderatus*, bter = *B. terrestris*.

**Supporting references**

Ellis JS, Knight ME, Goulson D (2005) Delineating species for conservation using mitochondrial sequence data: the taxonomic status of two problematic *Bombus* species (Hymenoptera: Apidae). *Journal of Insect Conservation*, **9**, 75-83.

Estoup A, Scholl A, Pouvreau A, Solignac M (1995) Monoandry and polyandry in bumble bees (Hymenoptera; Bombinae) as evidenced by highly variable microsatellites. *Molecular* *Ecology*, **4**, 89–93.

Estoup A, Solignac M, Cornuet JM, Goudet J, Scholl A (1996) Genetic differentiation of continental and island populations of *Bombus terrestris* (Hymenoptera: Apidae) in Europe. *Molecular Ecology*, **5**, 19–31.

Reber Funk C, Schmid-Hempel R, Schmid-Hempel P (2006) Microsatellite loci for *Bombus* spp. *Molecular Ecology Notes*, **6**, 83–86.

Stewart LC, Hale RJ, Hale ML (2010) Species-specific primers for the molecular identification of cryptic *Bombus* species in New Zealand. *Conservation Genetics*, **11**, 1207-1209.

Stolle E, Rohde M, Vautrin D, Solignac M, Schmid-Hempel P, Schmid-Hempel R, Moritz RFA (2009) Novel microsatellite DNA loci for *Bombus* *terrestris* (Linnaeus, 1758). *Molecular Ecology Resources*, **9**, 1345–1352.

Wang J (2004) Sibship reconstruction from genetic data with typing errors. *Genetics*, **166**, 1963–1979.
